# CNT@TiO_2_ nanohybrids for high-performance anode of lithium-ion batteries

**DOI:** 10.1186/1556-276X-8-499

**Published:** 2013-11-22

**Authors:** Zhenhai Wen, Suqin Ci, Shun Mao, Shumao Cui, Zhen He, Junhong Chen

**Affiliations:** 1 Department of Mechanical Engineering, University of Wisconsin-Milwaukee, 3200 North Cramer Street, Milwaukee, WI 53211, USA; 2 Department of Civil and Environmental Engineering, Virginia Polytechnic Institute and State University, Blacksburg, VA 24061, USA

**Keywords:** TiO_2_, Carbon nanotubes, Nanohybrids, Anode, Lithium ion batteries

## Abstract

This work describes a potential anode material for lithium-ion batteries (LIBs), namely, anatase TiO_2_ nanoparticle-decorated carbon nanotubes (CNTs@TiO_2_). The electrochemical properties of CNTs@TiO_2_ were thoroughly investigated using various electrochemical techniques, including cyclic voltammetry, electrochemical impedance spectroscopy, galvanostatic cycling, and rate experiments. It was revealed that compared with pure TiO_2_ nanoparticles and CNTs alone, the CNT@TiO_2_ nanohybrids offered superior rate capability and achieved better cycling performance when used as anodes of LIBs. The CNT@TiO_2_ nanohybrids exhibited a cycling stability with high reversible capacity of about 190 mAh g^-1^ after 120 cycles at a current density of 100 mA g^-1^ and an excellent rate capability (up to 100 mAh g^-1^ at a current density of 1,000 mA g^-1^).

## Background

The use of limited fossil fuel resources and their negative impact on the environment are significant challenges facing world economies today, creating an urgent demand for new technologies that enable high efficiencies in energy harvesting, conversion, and storage devices [1,2]. Various technologies, including fuel cells, batteries, solar cells, and capacitors, show great promise to significantly reduce carbon footprints, decrease reliance on fossil fuels, and develop new driving forces for economic growth [3,4]. Lithium-ion batteries (LIBs) have been regarded as one of the most promising energy storage technologies for various portable electronics devices [5], and one of the key goals in developing LIBs systems is to design and fabricate functional electrode materials that can lower costs, increase capacity, and improve rate capability and cycle performance [6-9].

It has been extensively reported that TiO_2_ is a promising candidate to compete with commercial graphite anode for LIBs due to its multiple advantages of high abundance, low cost, high Li-insertion potential (1.5 to 1.8 V vs. Li^+^/Li), structural stability, and excellent safety during cycling [10]. Practical applications of TiO_2_ in LIBs, however, face significant challenges of poor electrical conductivity and low chemical diffusivity of Li, which are two key factors for the lithium insertion-deinsertion reaction. Therefore, it is highly desirable to develop reliable strategies to advance electrical conductivity and Li^+^ diffusivity in TiO_2_[11,12]. In fact, continued breakthroughs have been made in the preparation and modification of TiO_2_-based nanomaterials for high performance energy conversion and storage devices [13,14].

It is generally acknowledged that there are three routes available to tune the properties of TiO_2_ for its corresponding applications: (1) preparation of TiO_2_-based nanostructures with specific morphology [11]. For example, TiO_2_-based nanorods were reported to show enhanced rate capability and improved stability as electrodes in LIBs due to their one-dimensional (1D) structure and high surface area [15,16]. (2) Synthesis of TiO_2_ nanocrystals with specific crystal surface orientations [17]. It was reported that TiO_2_-based nanocubes dominated by (001) planes had much higher catalytic activity for photo-degradation of organic dyes than the conventional TiO_2_ with mixed crystallographic facets [18,19]. (3) Fabricating TiO_2_-based nanohybrids with other functional materials. Carbon nanostructures, such as carbon nanotubes (CNTs) and graphene, are the most appealing functional materials for improving the performance of TiO_2_ nanostructures due to their unique structure, excellent electrical conductivity, high stability, and great mechanical properties [20,21].

We recently developed a convenient procedure to synthesize TiO_2_ nanoparticle-decorated CNT hybrid structures (CNTs@TiO_2_) through annealing treatment of carbonaceous polymer-modified CNTs with adsorbed Ti^4+^. The as-prepared CNT@TiO_2_ nanocomposites exhibit multiple favorable features, such as excellent electrical conductivity and considerable high surface area, which make them to be potentially used for promising electrode material of electrochemical energy storage and conversion devices. We systematically investigated the electrochemical properties of CNT@TiO_2_ nanohybrids as anodes of LIBs, and demonstrated that the unique properties of both CNTs and TiO_2_ can merge well in the CNT@TiO_2_ nanohybrids with synergetic effects. In this way, the CNTs@TiO_2_ can potentially address the intrinsic issues associated with TiO_2_ anodes in LIBs, namely poor electrical conductivity and low chemical diffusivity of Li ions, and thus significantly improve performance in term of capacity, cycle performance, and rate capability.

## Methods

### Materials and synthesis

All chemicals were purchased from Sigma-Aldrich (St. Louis, MO, USA) and used without further purification, except CNTs (200 nm in diameter) which were purchased from Carbon Nanotechnologies, Inc. (Sunnyvale, CA, USA). CNTs@TiO_2_ were prepared through a modified route reported previously [22]. Typically, 0.15-g CNTs were completely mixed with a 60-ml glucose solution (0.5 mg/ml) under sonication. The mixed turbid liquid was then placed in a 100-ml Teflon-lined stainless steel autoclave and heated at 180°C for 5 h. Next, 0.2 g of the product after centrifuging and drying, namely carbonaceous polymer-modified CNTs (CNTs@C_polymer_), was then dispersed in 15 ml ethanol with the addition of 1 ml of titanium isopropoxide (TIP, 97%) under vigorous agitation. After centrifuging and drying, the solid products were then calcined at 400°C and exposed in an air atmosphere to evolve into CNTs@TiO_2_. Powder X-ray diffraction (XRD) was conducted on a Scintag XDS 2000 X-ray powder diffractometer (Scintag Inc., Santa Clara, CA, USA) using monochromatized CuKα as radiation (*λ* = 1.5418 Å); the data were collected by scanning angles (2*θ*) from 20° to 60°. N_2_ adsorption-desorption experiments were tested at 77 K by a Quantachrome autosorb gas-sorption system (Boynton Beach, FL, USA). The morphologies of the as-prepared samples were observed using a Hitachi (H 9000 NAR, Tokyo, Japan) transmission electron microscope (TEM) and a Hitachi S-4800 scan electron microscope (SEM).

### Characterization

The working electrode of LIB was prepared by compressing a mixture of active materials (80%), acetylene black (10%), and polyvinylidene fluoride (10%) as a binder dissolved in 1-methyl-2-pyrrolidinone solution onto a copper foil. The pellet was dried in vacuum at 120°C for 10 h and then assembled into a coin cell in an Ar-protected glove box. The electrolyte solution was 1 M LiPF_6_ dissolved in a mixture of ethylene carbonate (EC) and dimethyl carbonate (DMC), with a volume ratio of EC/DMC = 4:6. Galvanostatic cycling experiments were conducted to measure the electrode activities using a Maccor battery tester system (Tulsa, OK, USA) at room temperature. Cyclic voltammograms (CVs) were carried out with three-electrode cells and recorded from 3.0 to 1.0 V at a scan rate of 0.1 mV s^-1^ using a CHI 600 electrochemical station (CHI Inc., Austin, TX, USA). Discharge–charge curves were recorded at fixed voltage limits between 3.0 and 1.0 V at various current densities. The specific capacity was calculated based on the total mass of the active materials. Electrochemical impedance spectroscopy (EIS) measurements were carried out at the open-circuit voltage state of fresh cells using a CHI600 (Austin, TX, USA) electrochemical workstation. The impedance spectra were recorded potentiostatically by applying an AC voltage of 5-mV amplitude over a frequency range from 100 kHz to 5 mHz.

## Results and discussion

**Figure 1:**
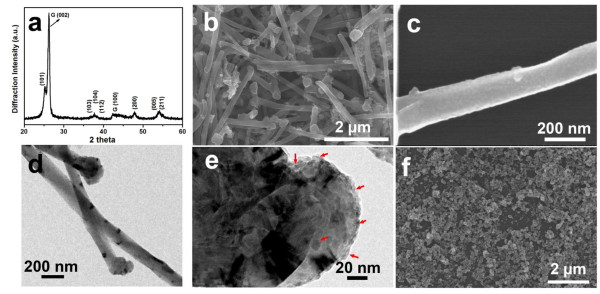
**XRD patterns and SEM, TEM, and HRTEM images of the hybrid CNTs@TiO**_**2**_**.** XRD patterns **(a)** and SEM image **(b)** of the CNT@TiO_2_ hybrids, SEM image **(c)** of a single CNT@TiO_2_ hybrid, TEM **(d)** and HRTEM **(e)** images of the tip of a CNT@TiO_2_ hybrid with red arrows indicating TiO_2_ nanoparticles, and SEM image **(f)** of TiO_2_ nanoparticles prepared through a sol–gel method.

The crystalline structure, morphology, and nanostructure of the products were firstly investigated using XRD, SEM, and TEM, as shown in Figure 1. Figure 1a shows the XRD pattern of the CNTs@TiO_2_, which shows typical peaks that can be well assigned to anatase TiO_2_ with characteristic peaks of CNTs, indicating the successful decoration of anatase TiO_2_ nanoparticles on CNTs. Figure 1b exhibits the typical SEM image of the as-prepared CNTs@TiO_2_, demonstrating that the samples have a 1D structure with an average diameter of around 200 nm. Figure 1c presents the SEM image of one single CNT@TiO_2_; one can observe a large number of nanoparticles uniformly decorated on the surface of the nanofiber, which stands in sharp contrast to the carbonaceous modified CNT with a relative smooth surface (Additional file 1: Figure S1). The TiO_2_-decorated CNTs were additionally confirmed by a typical TEM image (Figure 1d). Figure 1e shows the TEM image recording the nanostructure of the tip of the CNTs@TiO_2_; it was revealed that the size of TiO_2_ nanoparticles on the CNTs is around 5 ~ 10 nm. For comparison, we prepared TiO_2_ nanoparticles with an average diameter of 50 nm through a sol–gel method (Figure 1f).

**Figure 2:**
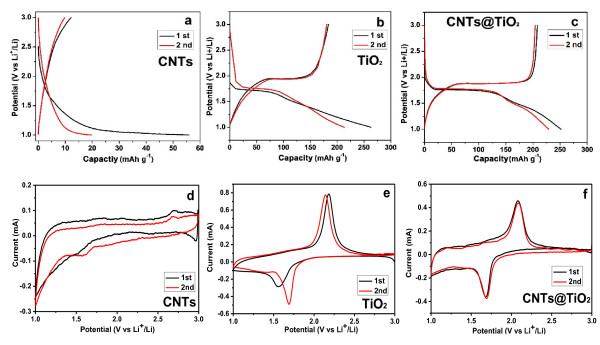
**The first two charge/discharge profiles and CV curves.** CNTs **(a)**, TiO_2_ nanoparticles **(b)**, and CNTs@TiO_2_**(c)** LIB anodes at a current density of 100 mA g^-1^. The initial two cyclic voltammograms of CNTs **(d)**, TiO_2_**(e)**, and CNTs@TiO_2_**(f)**.

The present CNTs@TiO_2_ feature a favorable porous structure and improved electrical conductivity, which are attractive for addressing the existing issues for TiO_2_ as anodes of LIBs; therefore, we systematically investigated the electrochemical performance of the CNTs@TiO_2_ as anode of LIBs. We first applied the techniques of galvanostatic charge/discharge and CV to compare and study the electrochemical properties of lithium insertion/deinsertion in half-cells based on CNT, TiO_2_, and CNT@TiO_2_ materials. Figure 2a,b,c and Figure 2d,e,f display the initial two charge–discharge profiles and CV curves for the CNT, TiO_2_, and CNT@TiO_2_ electrodes, respectively. The initial two charge–discharge profiles are generally consistent with the corresponding CV results. For CNTs, there is no pronounced peak in the range of 1.0 to 3.0 V with a remarkable discharge capacity loss from 55 mAh g^-1^ in the first cycle to 20 mAh g^-1^ in the second cycle. In contrast, both TiO_2_ and CNT@TiO_2_ electrodes show a discharge plateau at around 1.70 V and a charge plateau at about 1.90 V in the first cycle, which is basically consistent with those reported previously [20,21]. In particular, the TiO_2_ electrode exhibits a pronounced capacity loss of 20.0% in the second discharge process, while the CNT@TiO_2_ electrode only shows a capacity loss of less than 10.0% in the initial two cycles. As expected, there is a pair of peaks in the CV curves of the TiO_2_ and CNT@TiO_2_ electrodes, namely, the cathodic peak at 1.69 V and the anodic peak at 2.08 V, corresponding with the reversible biphasic transition between the tetragonal anatase and orthorhombic Li_*x*_TiO_2_, respectively (Equation 1).

(1) $$x{\mathrm{Li}}^{+}+xe^{-}+{\text{TiO}}_{2}↔{\mathrm{Li}}_{x}{\text{TiO}}_{2}\;\left(0\leq x\leq 1.0\right)$$

There is an observable decrease of cathodic current in the second CV compared with the first CV for the TiO_2_ electrode, which agrees with the previous report on TiO_2_ anode materials and can be attributed to the irreversible lithium insertion-deinsertion reaction, indicating a large capacity loss during the first two cycles. The CNTs@TiO_2_, however, only display a small change during the initial two CVs, suggesting a small capacity loss in the initial two cycles.

**Figure 3:**
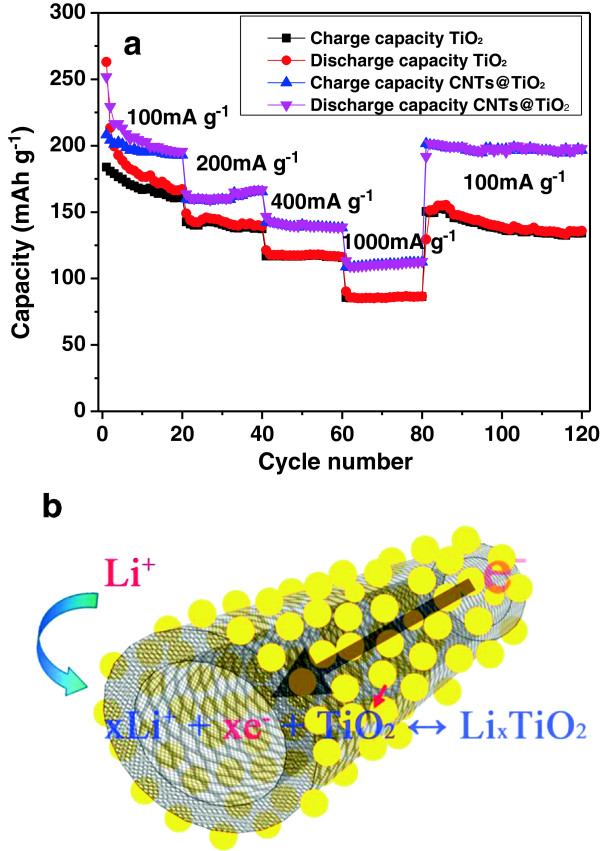
**Cyclic performance, rate capability, and scheme of Li**^**+ **^**insertion/deinsertion reaction.** Cyclic performance and rate capability of TiO_2_ and CNTs@TiO_2_ at current densities of 100, 200, 400, and 1,000 mA g^-1^**(a)**, and schematic illustration of the Li^+^ insertion/deinsertion reaction in CNT@TiO_2_ nanohybrids **(b)**.

Figure 3a compares the cycling performance and the rate capability between the TiO_2_ and CNTs@TiO_2_. The CNTs@TiO_2_ show significantly improved performance in terms of the capacity (except the first discharge capacity), rate capability, and stability. First, the CNTs@TiO_2_ showed a remarkable improvement in cycling performance compared with TiO_2_. The CNTs@TiO_2_ delivered a specific capacity of 251.9 mAh/g in the first cycle at a current density of 100 mA g^-1^. This value is slightly lower than the corresponding capacity of the TiO_2_ (263.0 mAh/g); however, the CNTs@TiO_2_ discharged a higher capacity than TiO_2_ in the following cycle. One can observe that the discharge capacity gradually decreased in the initial several cycles for both CNTs@TiO_2_ and TiO_2_. The CNT@TiO_2_ electrode achieved a stable capacity of around 195.5 mAh/g in the tenth cycle, while the TiO_2_ showed a continuous decrease, even in the initial 20 cycles. In fact, when the current density was switched back to 100 mA g^-1^ in the 81st cycle, the CNTs@TiO_2_ reached a reversible capacity of around 191.0 mAh g^-1^ and maintained this capacity in the subsequent cycles, while the TiO_2_ discharged a corresponding capacity of 163.3 mAh g^-1^ and showed a slow decrease with the continuous cycling. In addition, the CNTs@TiO_2_ also exhibited a greatly improved rate performance compared with TiO_2_, with varying current densities from 100 to 1,000 mA g^-1^. For instance, the CNTs@TiO_2_ maintained a capacity of 110 mAh g^-1^ at a current density of as high as 1,000 mA g^-1^, while the TiO_2_ only had a capacity of around 85 mAh g^-1^ under this current density. It should be noted that the CNTs@TiO_2_, as an anode of LIBs, also show improved electrochemical performance compared with the TiO_2_ nanostructures reported previously [23-25], signifying that the as-designed CNTs@TiO_2_ show great promise to advance electrochemical performance. In addition, the CNTs@TiO_2_ can compete with or outperform the TiO_2_/CNT composites reported previously in terms of capacity and cycling performance [26,27]. For instance, the CNTs@TiO_2_ still retained a specific capacity of about 190 mAh g^-1^ at a current density of 100 mA g^-1^[28], which shows a remarkable contrast to the blended TiO_2_/CNT that only retained a capacity of about 170 mAh g^-1^ at the same current density.

Figure 3b schematically illustrates the Li^+^ insertion/deinsertion in CNT@TiO_2_ nanohybrids and demonstrates advantages of the high electrical conductivity and facile transport of Li^+^ in CNT@TiO_2_ nanohybrids. The improved electrochemical performance in CNT@TiO_2_ nanohybrids can be attributed to the following factors: above all, the TiO_2_ nanoparticles were uniformly decorated on the surface of one-dimensional CNTs, which offered excellent flexibility and enough space for alleviating the effects of electrode degradation and volume change upon cycling. Additionally, the large surface area (109.9 m^2^ g^-1^) and suitable pore size (11.5 nm) in CNTs@TiO_2_ can facilitate the transport of electrolytes and Li^+^ on the interface of electrodes, leading to good rate capability. Furthermore, the electrical conductivity, thanks to the CNT's core, is expected to be greatly enhanced, which can significantly decrease the capacity loss from Ohmic resistance.

**Figure 4:**
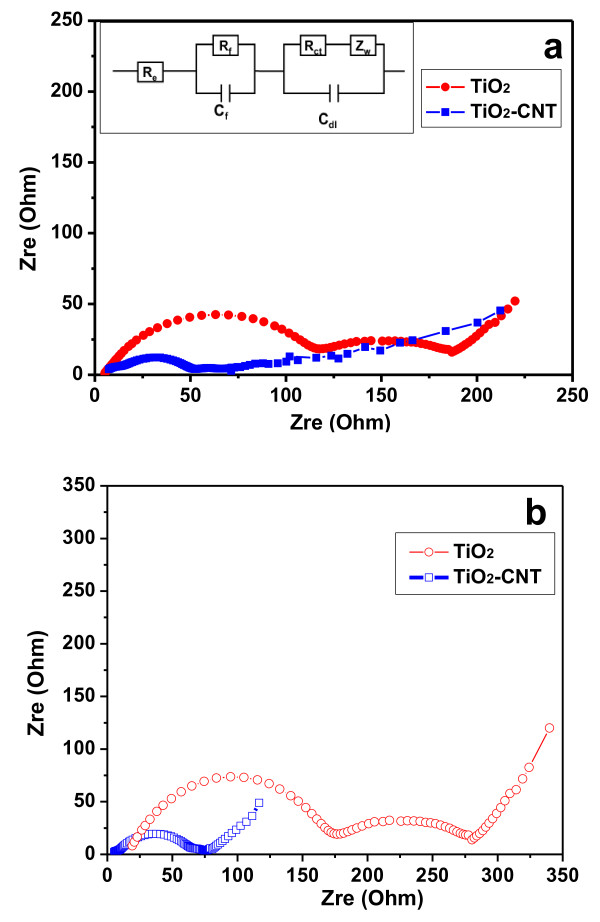
**Nyquist curves of the LIB with TiO**_**2 **_**and CNTs@TiO**_**2 **_**as the working electrode.** Before cycling **(a)** and after 120 charge–discharge cycles **(b).**

 The EIS measurements were carried out to investigate the resistance associated with the TiO_2_ and the CNTs@TiO_2_. Figure 4 shows the Nyquist plots recorded for the TiO_2_ and the CNTs@TiO_2_, respectively, which typically consists of a high-frequency semicircle corresponding with the charge transfer resistances (*R*_ct_). The Nyquist data were then fitted to a hypothetical equivalent circuit (inset of Figure 4a) to evaluate the *R*_ct_ and the resistance of the film formed on the electrode surface (*R*_f_). It was revealed that the *R*_ct_ and *R*_f_ for the CNTs@TiO_2_ were 48.8 and 21.3 Ω, respectively, much lower than the corresponding *R*_ct_ (117.95 Ω) and *R*_f_ (72.0 Ω) for the TiO_2_ electrode, indicating that the CNTs@TiO_2_ have a significantly lower overall impedance, which might be one of the key factors responsible for the improved electrochemical performance of the CNTs@TiO_2_. We further investigated the impedance change after cycling; it was revealed that the TiO_2_/CNT only shows a slight change in impedance spectroscopy, while the TiO_2_ exhibits an evident change in impedance spectroscopy after 120 cycles (Figure 4b). These results additionally confirmed that the former can well maintain the high conductivity upon cycling.

## Conclusion

In summary, we demonstrated the electrochemical properties of the nanohybrids of TiO_2_ nanoparticle-decorated CNTs as an anode of lithium-ion batteries. The CNT@TiO_2_ hybrids showed better electrochemical performance than the pure TiO_2_ nanoparticles with regard to specific capacity (except the initial cycle), rate capability, and cycling stability. The improved electrochemical performance can be ascribed to the synergetic effects of combined properties, including the one-dimensional structure, high-strength with flexibility, excellent electrical conductivity, and large surface area.

## Competing interests

The authors declare that they have no competing interests.

## Authors' contributions

ZHW conducted synthetic and battery testing experiments, and drafted the manuscript. SQC conducted electrochemical test. SMC carried out TEM. SM carried out SEM. JHC and ZH conceived the study. All authors read and approved the final manuscript.

## Authors' information

ZHW obtained his Ph.D. from the Chinese Academy of Sciences in 2008. After working as a Humboldt postdoctoral research scholar at the Max-Planck Institute for Polymer Research in Germany. He started his postdoctoral research at the University of Wisconsin-Milwaukee (UWM). His research is primarily focused on electrochemical or photocatalytic energy storage and conversion. SQC worked as a lecturer at Nanchang Hangkong University in China after receiving her Ph.D. in Biochemical Engineering from the Institute of Process Engineering, Chinese Academy of Sciences. Currently, she is a postdoctoral researcher at the University of Wisconsin-Milwaukee and working on electrochemical analysis and electrocatalysis. SM received his Ph.D. in Mechanical Engineering from UWM in 2010 for the study of hybrid nanomaterials for biosensing applications. After graduation, he worked as a project director at NanoAffix Science, LLC for a hydrogen sensor project. He is currently a postdoctoral fellow at UWM. His research is focused on hybrid nanostructures (i.e., graphene/CNT with nanocrystals) for energy and environmental applications. SMC received his Ph.D. in Mechanical Engineering from UWM in 2013 and is currently a postdoctoral fellow at UWM. His research interests include synthesis of nanoparticles, synthesis of nanohybrids combining nanocarbons (graphene and carbon nanotubes) with nanoparticles, and developing environment and energy applications using nanomaterials. ZH is an associate professor of the Department of Civil and Environmental Engineering at Virginia Polytechnic Institute and State University. He received his B.E. degree from Tongji University, M.Sc. degree from the Technical University of Denmark, and Ph.D. from Washington University in St. Louis. He completed his postdoctoral training at the Mork Family Department of Chemical Engineering and Materials Science and the Department of Earth Sciences at the University of Southern California. Before joining VT, he was an assistant professor of civil engineering at UWM. His research focuses on the fundamental understanding of engineered systems for bioenergy production from wastes and development of bioelectrochemical systems for water and wastewater treatment. JHC received his B.E. degree in thermal Engineering from Tongji University, Shanghai, China, in 1995 and M.S. and Ph.D. degrees in Mechanical Engineering from the University of Minnesota, Minneapolis, MN, in 2000 and 2002, respectively. From 2002 to 2003, he was a postdoctoral scholar in Chemical Engineering at California Institute of Technology. He is currently a full Professor in the Department of Mechanical Engineering at UWM. His current research interests include carbon nanotube- and graphene-based hybrid nanomaterials, plasma reacting flows, and nanotechnology for sustainable energy and environment.

## Supplementary Material

Additional file 1: Figure S1 SEM image of the carbonaceous modified CNTs. Click here for file
