# Supplementary material for: CNT@TiO2 nanohybrids for high-performance anode of lithium-ion batteries
Source: Nanoscale Res Lett. 2013 Nov 22;8(1):499. doi: 10.1186/1556-276X-8-499 (PMC3874633; doi:10.1186/1556-276X-8-499)
Supplement: Additional file 1: Figure S1 — SEM image of the carbonaceous modified CNTs. [file 1556-276X-8-499-S1.doc]

**TOC for：CNTs@TiO_2_ Nanohybrids for High-performance Anode of Lithium-Ion Batteries**

*Zhenhai Wen,^a^  Suqin Ci,^a^ Shun Mao,^a^ Shumao Cui,^a^ Zhen He^b,*^ and Junhong Chen^a,*^*

^a^ Department of Mechanical Engineering, University of Wisconsin-Milwaukee, 3200 North Cramer Street, Milwaukee, Wisconsin 53211, USA

^b^ Department of Civil and Environmental Engineering, Virginia Polytechnic Institute and State University, Blacksburg, VA 24061, USA


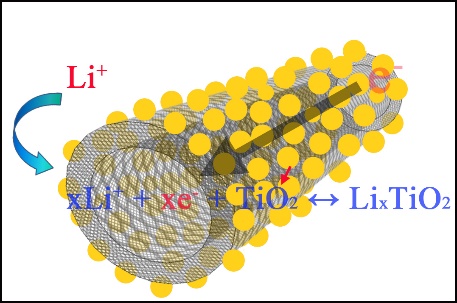


The anatase TiO_2_ nanoparticles-decorating carbon nanotubes (CNTs@TiO_2_) are synthesized, which displays a unique feature of having CNTs encapsulated inside and anatase TiO_2_ nanoparticles coating on the CNT surface. The as-developed CNTs@TiO_2_ nanohybrids offered superior rate capability and outstanding cycling performance as anodes of lithium-ion batteries (LIBs).

**Supporting Information for: CNTs@TiO_2_ Nanohybrids for High-performance Anode of Lithium-Ion Batteries**

*Zhenhai Wen,^a^  Suqin Ci,^a^ Shun Mao,^a^ Shumao Cui,^a^ Zhen He^b,*^ and Junhong Chen^a,*^*

^a^ Department of Mechanical Engineering, University of Wisconsin-Milwaukee, 3200 North Cramer Street, Milwaukee, Wisconsin 53211, USA

^b^ Department of Civil and Environmental Engineering, Virginia Polytechnic Institute and State University, Blacksburg, VA 24061, USA

**
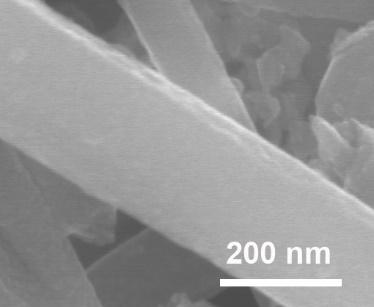
**

Figure S1. SEM image of the carbonaceous modified CNTs.
